# Supplementary material for: Membrane mediated mechanical stimuli produces distinct active-like states in the AT1 receptor
Source: Nat Commun. 2023 Aug 4;14:4690. doi: 10.1038/s41467-023-40433-4 (PMC10403497; doi:10.1038/s41467-023-40433-4)
Supplement: Supplementary file 1 — Supplementary Information [file 41467_2023_40433_MOESM1_ESM.pdf]

Supplementary Information

Membrane mediated mechanical stimuli produces distinct active-like states in the AT1 receptor

Bharat Poudel[1], Rajitha Rajeshwar T.[2], and Juan M. Vanegas[1,2,3*]

[1]Material Science graduate Program, The University of Vermont, Burlington, Vermont, 05405, USA
[2]Department of Physics, University of Vermont, Burlington, Vermont, 05405, USA
[3]Current Address: Department of Biochemistry and Biophysics, Oregon State University, Corvallis, OR, 97330, USA
[*]Correspondence: vanegasj@oregonstate.edu

SUPPLEMENTARY TABLES

SUPPLEMENTARY TABLE 1. System details for GROMOS simulations

System	Total Nr. of Atoms	Box dimensions (nm)	Nr. of Lipids	Nr. of Waters	Cl^-
DMPC + AT1R	99,132	10×10×11.8	300	27,388	13
POPC + AT1R	112,330	10.4×10.4×13.5	304	31,068	13
SOPC + AT1R	106,560	10.4×10.4×13.5	298	29,050	13
SOPC:SOPE + AT1R	90,885	9.5×9.5×13	300	23,789	13
SOPC + AT1R + AngII	106,662	10.4×10.4×13.5	298	29,050	13
SOPC + AT1R + S1I8	106,651	10.4×10.4×13.5	298	29,050	14
SOPC + AT1R + S1I8 +Nb	107,821	10.4×10.4×13.5	298	29,056	8
SOPC + AT1R + Nb	107,912	10.4×10.4×13.5	298	29,056	7
POPC + F309P/313P AT1R	82,319	10.4×10.4×10	304	21,071	13
SOPC + F309P/313P AT1R	83,329	10.5×10.5×11	298	21,313	13
SOPC:SOPE + F309P/313P AT1R	72,427	9.5×9.5×10	300	17,643	13

SUPPLEMENTARY TABLE 2. System details for CHARMM36 simulations

System	Total Nr. of Atoms	Box dimensions (nm)	Nr. of Lipids	Nr. of Waters	Cl^-	Na^+
POPC + AT1R	49,146	6.4×6.4×11.6	100	10,154	39	26
SOPC + AT1R	52,021	6.4×6.4×12.5	100	10,911	41	28
SOPC + AT1R + AngII	48,274	6.7×6.7×11.5	100	9,616	37	24

SUPPLEMENTARY FIGURES

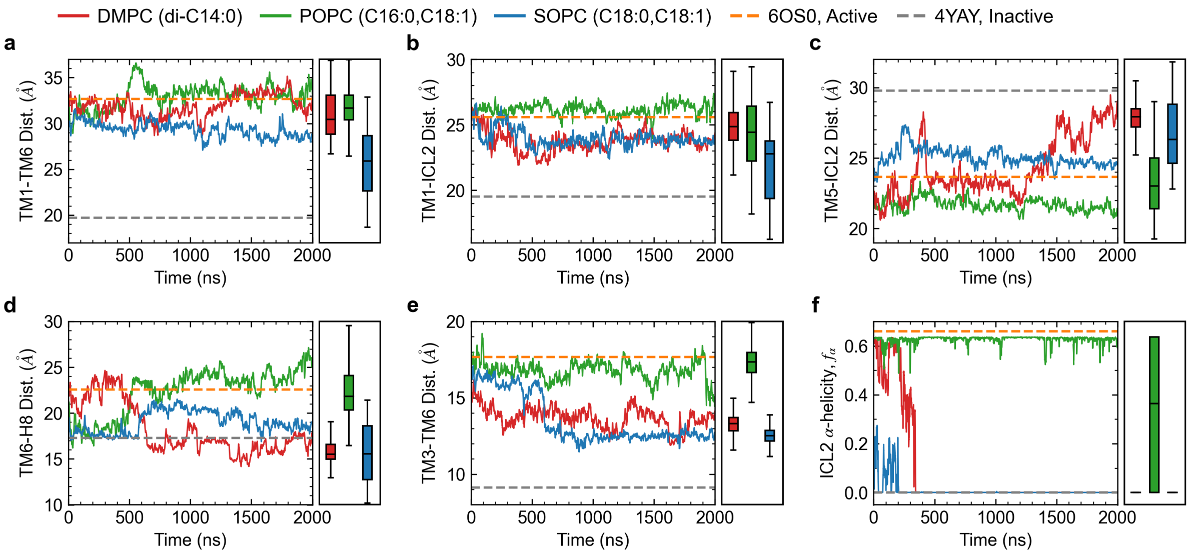

SUPPLEMENTARY FIGURE 1. Time evolution of apo AT1 receptor simulations (second replica) in PC membranes of varying chain lengths. Key structural features including the distances between TM1-TM6 (a), TM1-ICL2 (b), TM5-ICL2 (c), TM6-H8 (d) and TM3-TM6 (e), as well as the α-helicity of ICL2 (f) indicate that the active state is stable in the POPC (green lines) membrane, while thicker (SOPC, blue lines) or thinner (DMPC, red lines) membranes promote inactivation. Rectangular boxes on the right of each panel show box and whiskers plots including median, quartiles, and extrema of the combined data from the two replicas of each system over the last 500 ns (n = 2 independent simulations, 200,000 time points analyzed). Dashed grey and orange lines show values from crystal structures of the inactive receptor bound to a selective antagonist (4YAY) and active receptor bound to AngII (6OS0) respectively.

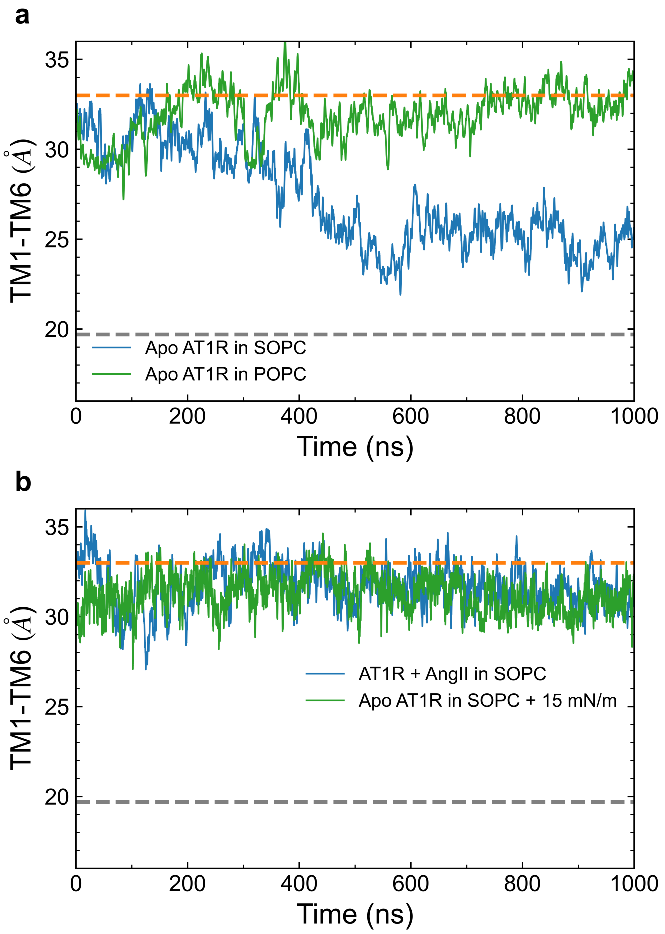

SUPPLEMENTARY FIGURE 2. Time evolution of the TM1-TM6 distance in the AT1 receptor in SOPC and POPC membrane simulations using the CHARMM36 forcefield. a) Apo AT1 receptor in SOPC and POPC. b) AT1 receptor in SOPC bound to angiotensin II and apo AT1 receptor in SOPC with a membrane tension of 15 mN/m. Dashed grey and orange lines show values from crystal structures of the inactive receptor bound to a selective antagonist (4YAY) and active receptor bound to AngII (6OS0) respectively.

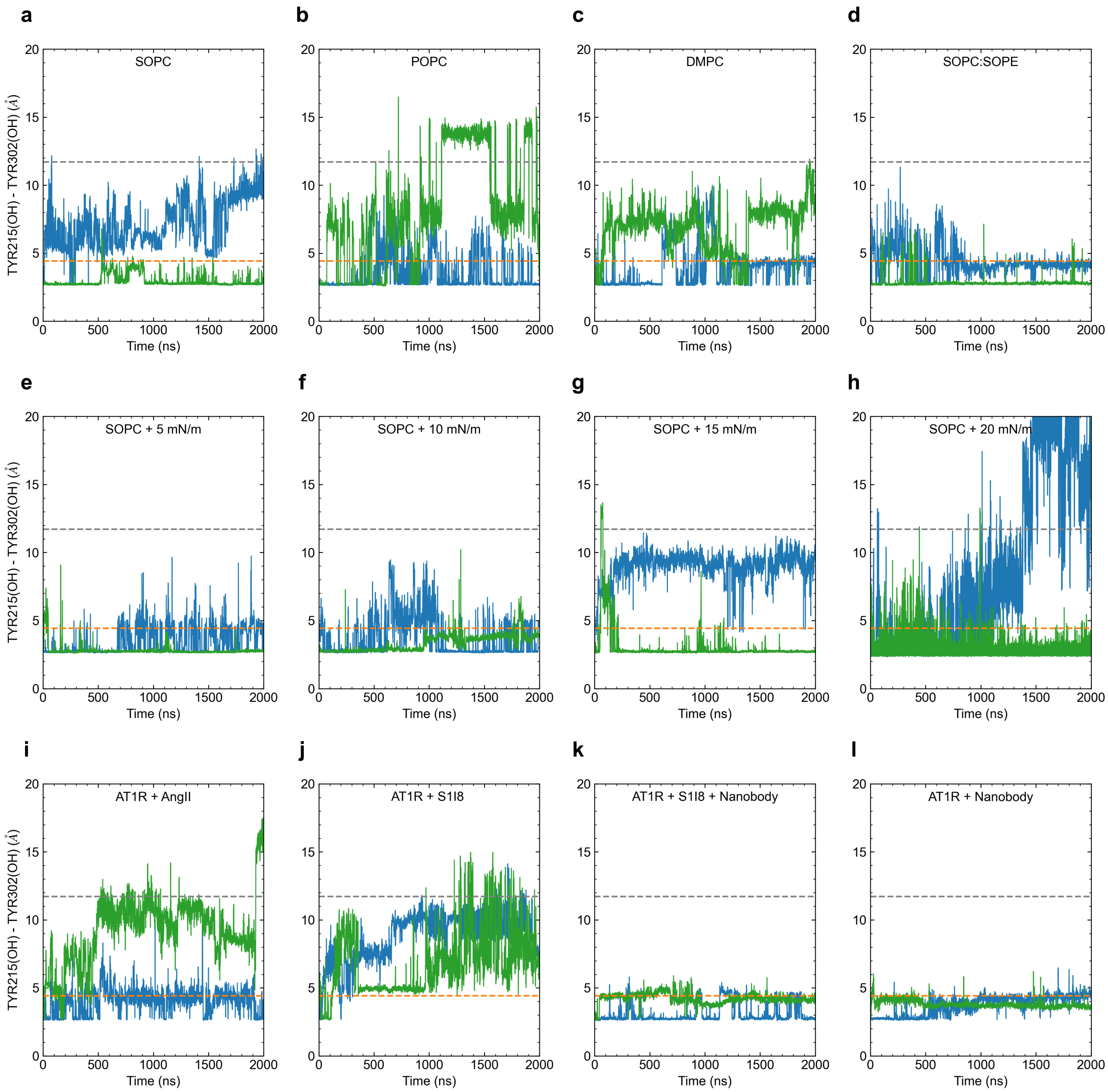

SUPPLEMENTARY FIGURE 3. Orientation of the conserved NPxxY motif in TM7 monitored by the distance between the hydroxyl oxygens of Y215$^{5.58}$ and Y302$^{7.53}$. Top row panels (a-b) show distances for the SOPC, POPC, DMPC, and SOPC:SOPE systems. Middle panels (e-h) show distances for SOPC bilayers under tension. Bottom row panels (i-l) show distances for systems with the AT1 receptor bound to agonists and/or nanobody. The blue and green lines represent the two simulated replicas.

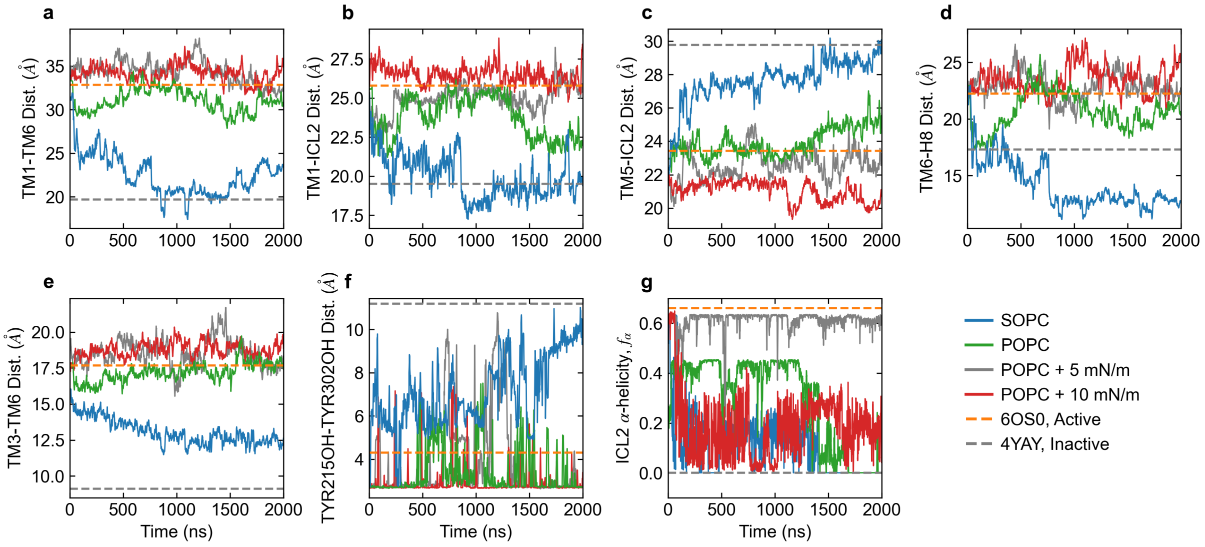

SUPPLEMENTARY FIGURE 4. Stability of the AT1 receptor in POPC membranes under tension (5 and 10 mN/m) starting from an active configuration. Panels a-f show the average intra-protein distances between TM1-TM6, TM1-ICL2, TM5-ICL2, TM6-H8, and TM3-TM6 as well as the α-helicity of ICL2 respectively. Data for SOPC without tension shown for comparison. Dashed grey and orange lines show values from crystal structures of the inactive receptor bound to a selective antagonist (4YAY) and active receptor bound to AngII (6OS0) respectively. Data taken from a single simulation.

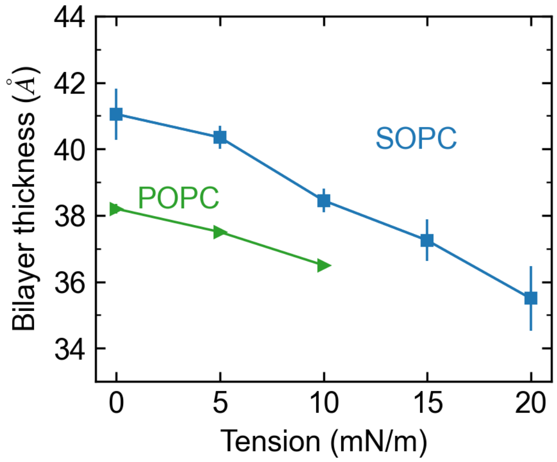

SUPPLEMENTARY FIGURE 5. Bilayer thickness for POPC (green triangles) and SOPC (blue squares) membranes under tension. Bilayer thickness was computed by measuring the peak-to-peak distance of the average bilayer mass density over the last 500 ns of the two replicas (n = 2 independent simulations, 200,000 time points analyzed). Error bars show the standard deviation from the mean.

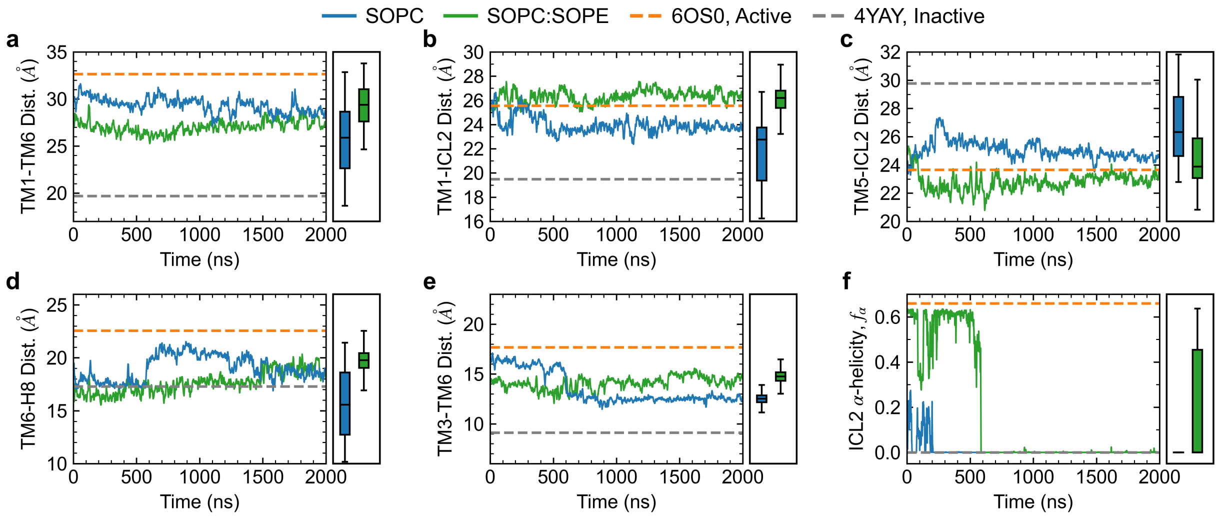

SUPPLEMENTARY FIGURE 6. Time evolution of apo AT1 receptor simulations in SOPC and SOPC:SOPE (1:1) membranes for the second replica. Key structural features including the distances between TM1-TM6 (a), TM1-ICL2 (b), TM5-ICL2 (c), TM6-H8 (d) and TM3-TM6 (e), as well as the α-helicity of ICL2 (f) indicate that the active state is stable in the SOPC:SOPE (green lines) membrane, while the pure SOPC membrane promotes inactivation. Rectangular boxes on the right of each panel show box and whiskers plots including median, quartiles, and extrema of the combined data from the two replicas of each system over the last 500 ns (n = 2 independent simulations, 200,000 time points analyzed). Dashed grey and orange lines show values from crystal structures of the inactive receptor bound to a selective antagonist (4YAY) and active receptor bound to AngII (6OS0) respectively.

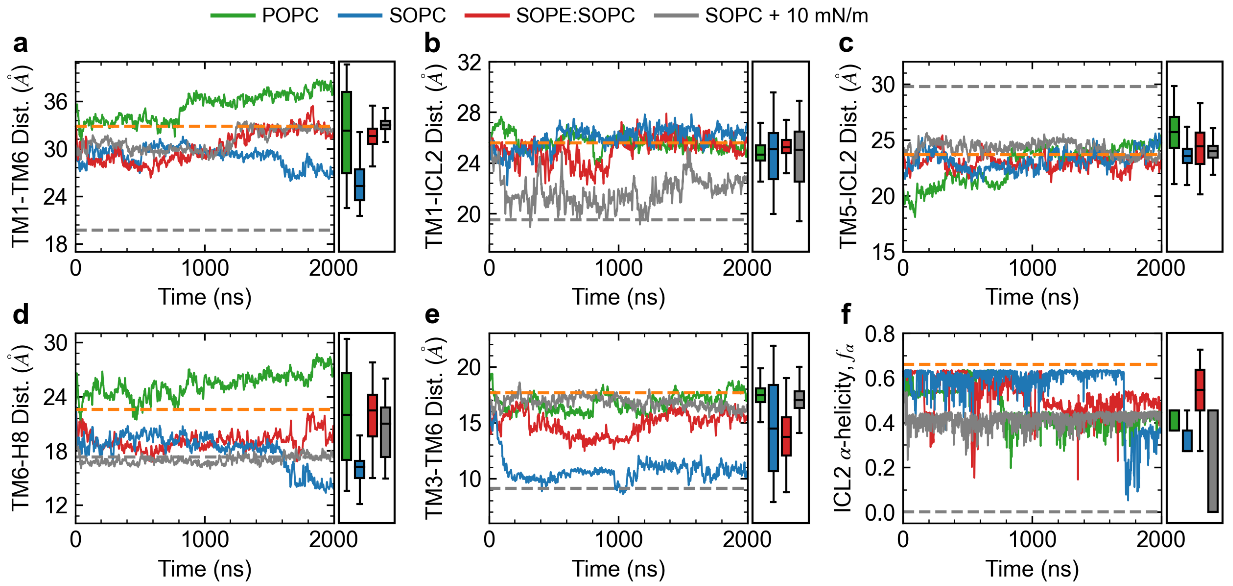

SUPPLEMENTARY FIGURE 7. Time evolution of double mutant F309P/313P AT1 receptor (apo) simulations in various membranes for replica 1. Panels a-f show the average intra-protein distances between TM1-TM6, TM1-ICL2, TM5-ICL2, TM6-H8, and TM3-TM6 as well as the α-helicity of ICL2 respectively. Data for SOPC shown in blue, SOPC with 10 mN/m tension in grey, POPC in green, and SOPC:SOPE in red. Rectangular boxes on the right of each panel show box and whiskers plots including median, quartiles, and extrema of the two replicas of each system over the last 500 ns (n = 2 independent simulations, 200,000 time points analyzed). Dashed grey and orange lines show values from crystal structures of the inactive receptor bound to a selective antagonist (4YAY) and active receptor bound to AngII (6OS0) respectively.

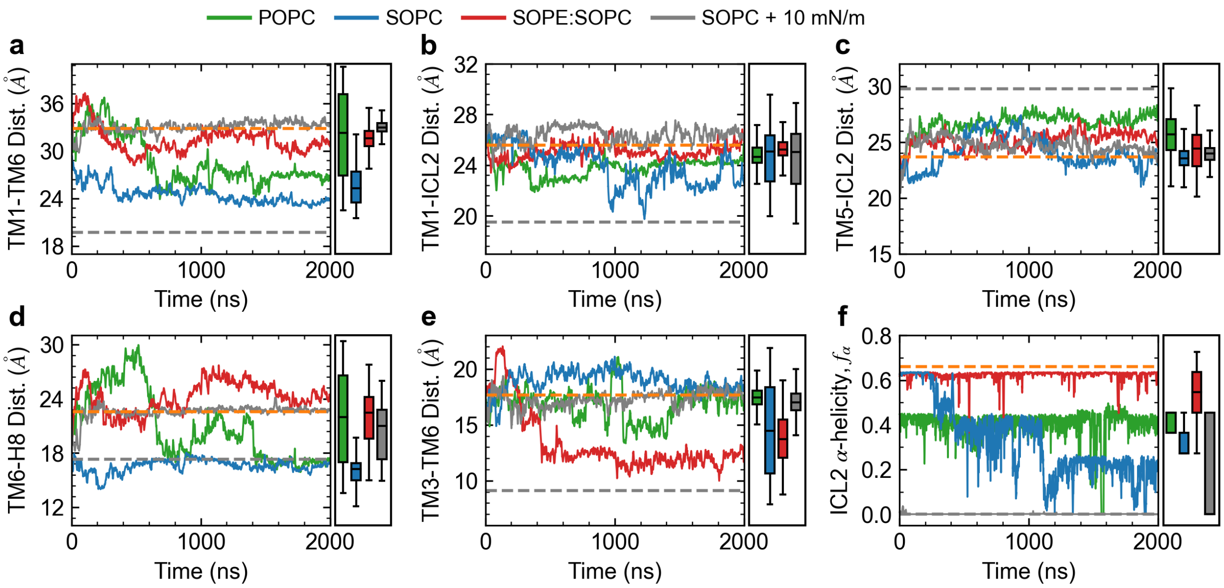

SUPPLEMENTARY FIGURE 8. Time evolution of double mutant F309P/313P AT1 receptor (apo) simulations in various membranes for replica 2. Panels a-f show the average intra-protein distances between TM1-TM6, TM1-ICL2, TM5-ICL2, TM6-H8, and TM3-TM6 as well as the α-helicity of ICL2 respectively. Data for SOPC shown in blue, SOPC with 10 mN/m tension in grey, POPC in green, and SOPC:SOPE in red. Rectangular boxes on the right of each panel show box and whiskers plots including median, quartiles, and extrema of the two replicas of each system over the last 500 ns (n = 2 independent simulations, 200,000 time points analyzed). Dashed grey and orange lines show values from crystal structures of the inactive receptor bound to a selective antagonist (4YAY) and active receptor bound to AngII (6OS0) respectively.

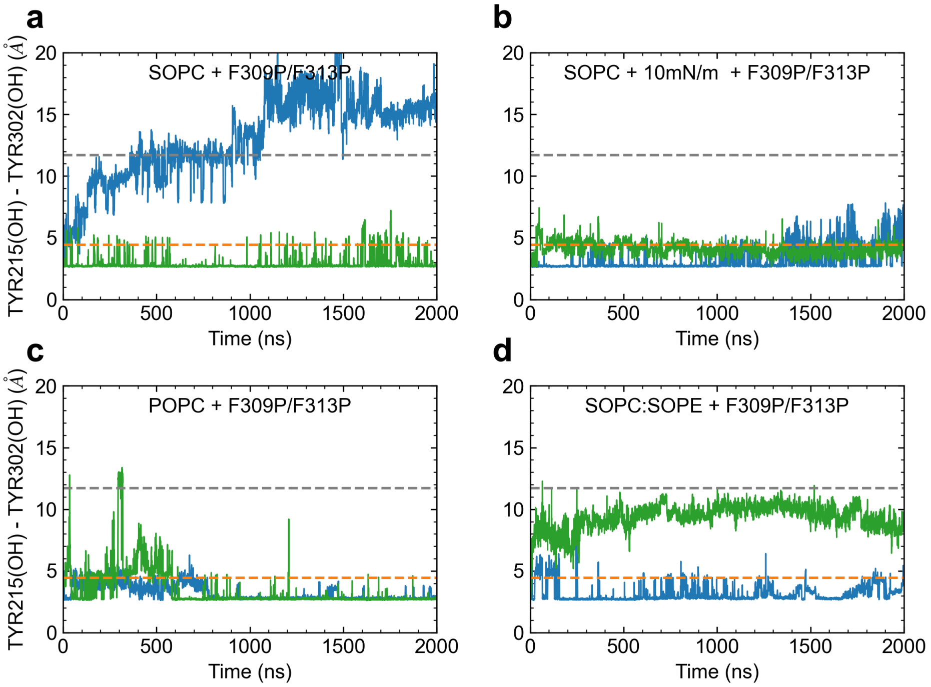

SUPPLEMENTARY FIGURE 9. Orientation of the conserved NPxxY motif in TM7 monitored by the distance between the hydroxyl oxygens of Y215$^{5.58}$ and Y302$^{7.53}$. Data for two replicas (blue and green) of the double-mutant F309P/F313P in SOPC (a), SOPC with 10 mN/m tension (b), POPC (c), and SOPC:SOPE (d).

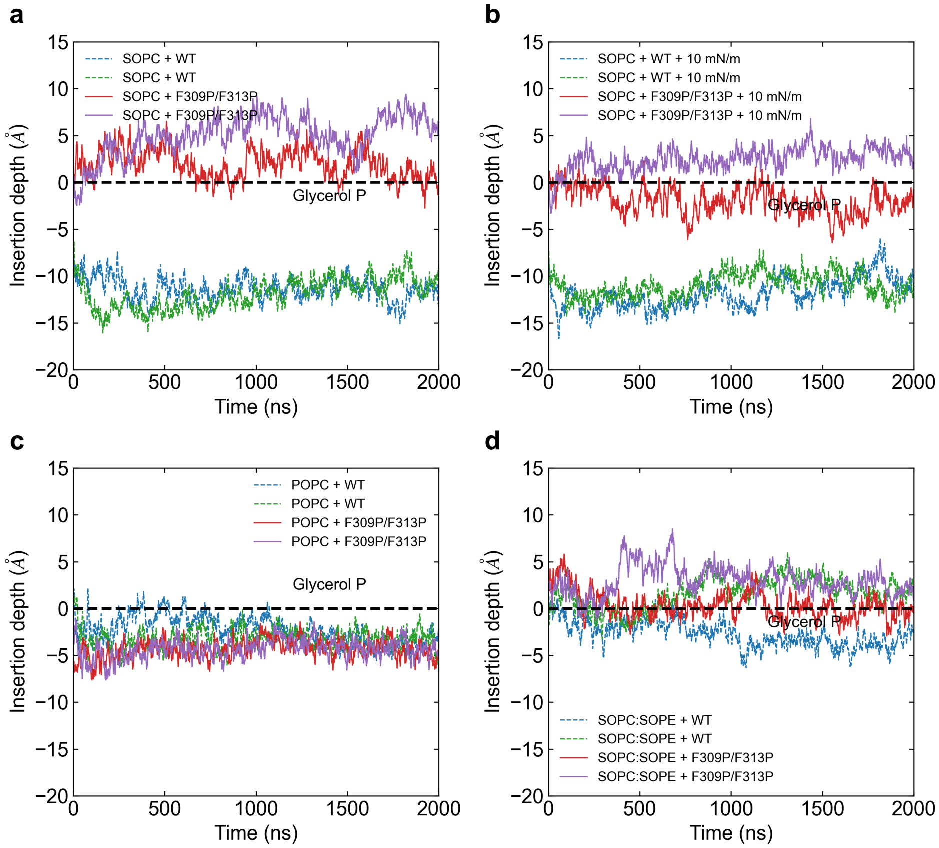

SUPPLEMENTARY FIGURE 10. Depth of insertion of H8 relative to the average position of the glycerol phosphate atoms on the cytoplasmic side of the membrane. Data shown for the apo WT (dashed lines) and apo double-mutant F309P/313P (solid lines) AT1 receptor in SOPC (a), SOPC with 10 mN/m tension (b), POPC (c), and SOPC:SOPE (d). Data from each replica shown in different colors. Negative values indicate movement of H8 into the hydrocarbon core, while positive values indicate movement toward the water interface.

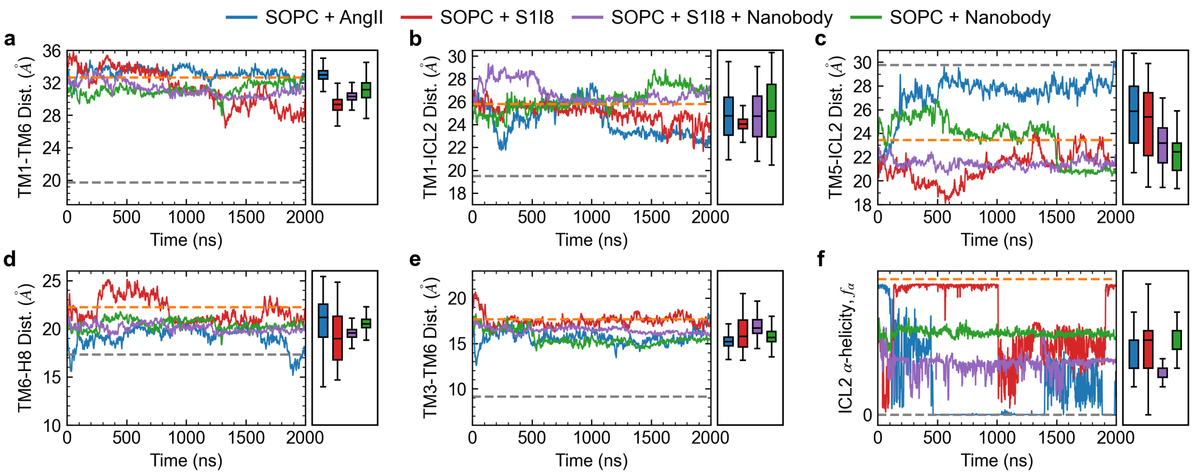

SUPPLEMENTARY FIGURE 11. Time evolution of AT1 receptor simulations (replica 2) in SOPC bound to the agonists AngII and S1I8 as well as the AT110i1 nanobody. Panels a-f show the AT1 receptor intra-protein distances between TM1-TM6, TM1-ICL2, TM5-ICL2, TM6-H8, and TM3-TM6, as well as the α-helicity of ICL2 respectively. Rectangular boxes on the right of each panel show box and whiskers plots including median, quartiles, and extrema of the combined data from the two replicas of each system over the last 500 ns (n = 2 independent simulations, 200,000 time points analyzed). Dashed grey and orange lines show values from crystal structures of the inactive receptor bound to a selective antagonist (4YAY) and active receptor bound to AngII (6OS0) respectively.

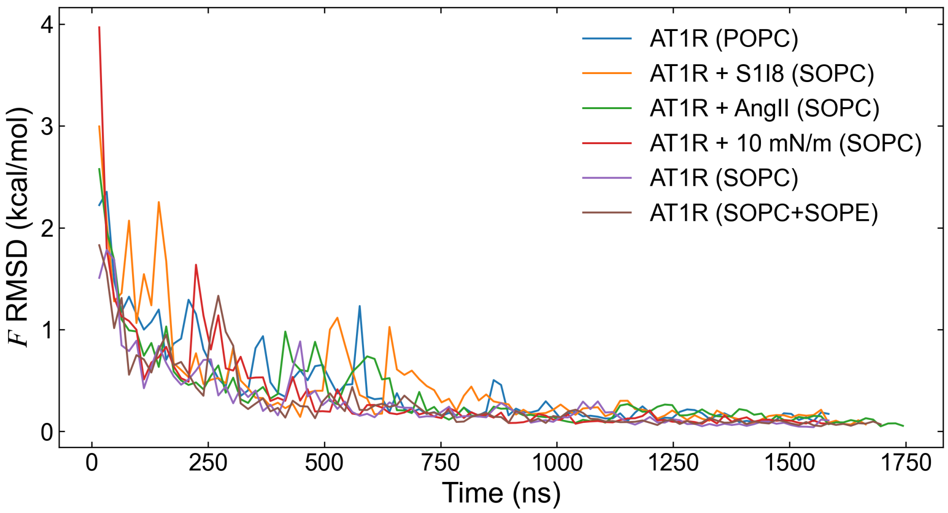

SUPPLEMENTARY FIGURE 12. Root-mean-squared deviation of the free energy landscape as a function of time for determining the convergence of the well-tempered metadynamics calculations.

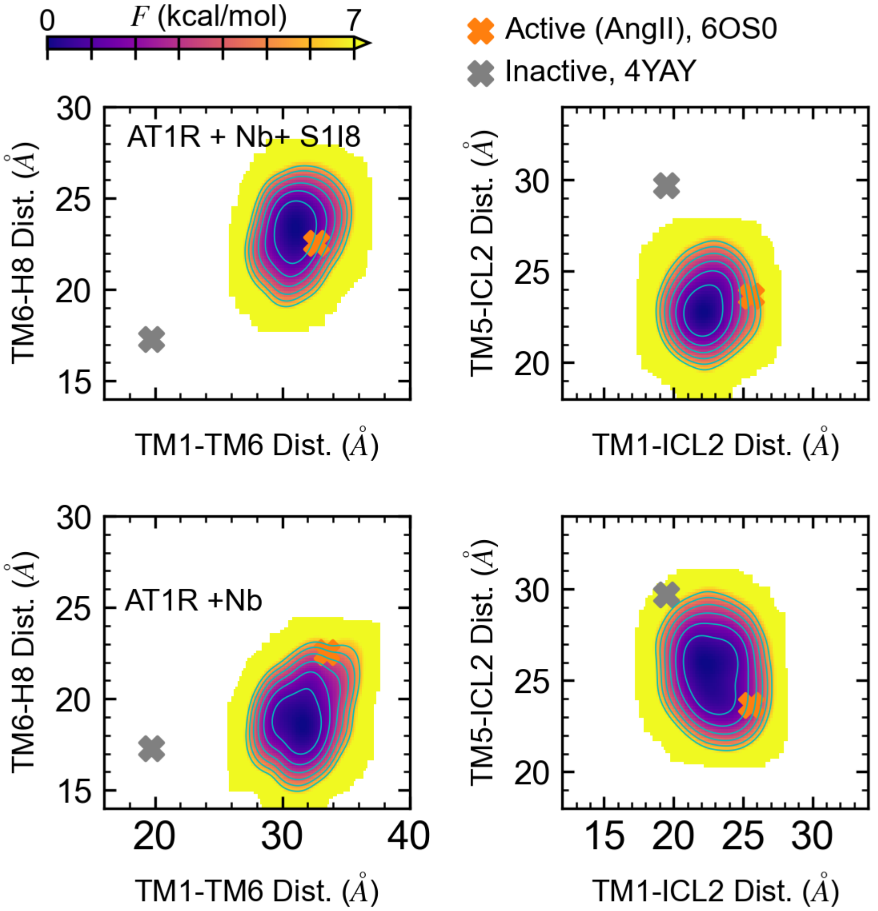

SUPPLEMENTARY FIGURE 13. Free energy estimation of AT1 receptor in SOPC membrane with a) S1I8 and nanobody (Nb) bound, and b) with only nanobody bound. Free energy estimated with LDT multiple-walker well-tempered metadynamics and plotted as two dimensional surfaces through histogram re-weighting using the TM1-TM6, TM6-H8, TM1-ICL2, and TM5-ICL2 distances (see Methods). Energy scale shown on top left with lowest values shown in dark blue and highest values shown in yellow (≥ 7 kcal/mol). Isocontour lines shown in cyan color drawn in 1 kcal/mol intervals. Grey markers show distance values from the inactive state crystal structure with receptor bound to a selective antagonist (PDBID: 4YAY) and orange markers show distance values from the active state crystal structure with bound AngII (PDBID: 6OS0).

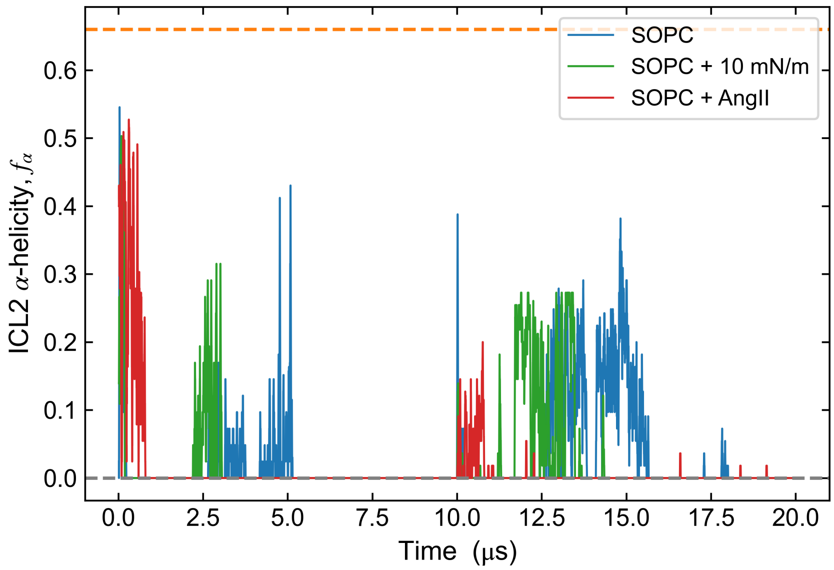

SUPPLEMENTARY FIGURE 14. α-helicity of ICL2 for selected Anton 2 simulations with the CHARMM36 FF. Dashed grey and orange lines show values from crystal structures of the inactive receptor bound to a selective antagonist (4YAY) and active receptor bound to AngII (6OS0) respectively.
