# Supplementary material for: Membrane mediated mechanical stimuli produces distinct active-like states in the AT1 receptor
Source: Nat Commun. 2023 Aug 4;14:4690. doi: 10.1038/s41467-023-40433-4 (PMC10403497; doi:10.1038/s41467-023-40433-4)
Supplement: Supplementary file 3 — Reporting Summary [file 41467_2023_40433_MOESM3_ESM.pdf]

nature portfolio

Corresponding author(s): Juan M. Vanegas

Last updated by author(s): Jun 28, 2023

Reporting Summary

Nature Portfolio wishes to improve the reproducibility of the work that we publish. This form provides structure for consistency and transparency in reporting. For further information on Nature Portfolio policies, see our Editorial Policies and the Editorial Policy Checklist.

Statistics

For all statistical analyses, confirm that the following items are present in the figure legend, table legend, main text, or Methods section.

n/a	Confirmed	
☐	☒	The exact sample size (n) for each experimental group/condition, given as a discrete number and unit of measurement
☐	☒	A statement on whether measurements were taken from distinct samples or whether the same sample was measured repeatedly
☐	☒	The statistical test(s) used AND whether they are one- or two-sided<br>*Only common tests should be described solely by name; describe more complex techniques in the Methods section.*
☐	☒	A description of all covariates tested
☐	☒	A description of any assumptions or corrections, such as tests of normality and adjustment for multiple comparisons
☐	☒	A full description of the statistical parameters including central tendency (e.g. means) or other basic estimates (e.g. regression coefficient) AND variation (e.g. standard deviation) or associated estimates of uncertainty (e.g. confidence intervals)
☒	☐	For null hypothesis testing, the test statistic (e.g. F, t, r) with confidence intervals, effect sizes, degrees of freedom and P value noted<br>*Give P values as exact values whenever suitable.*
☒	☐	For Bayesian analysis, information on the choice of priors and Markov chain Monte Carlo settings
☒	☐	For hierarchical and complex designs, identification of the appropriate level for tests and full reporting of outcomes
☒	☐	Estimates of effect sizes (e.g. Cohen's d, Pearson's r), indicating how they were calculated

Our web collection on statistics for biologists contains articles on many of the points above.

Software and code

Policy information about availability of computer code

Data collection	Molecular dynamics simulations were performed using the open source GROMACS simulation package version 2019. Free energy calculations were computed using a combination of the PLUMED plugin (v.2.5-2.7) and GROMACS 2019.
Data analysis	All plots in the manuscript were made with the Matplotlib python library v.3.3.3. Molecular models were made using the UCSF Chimera v.1.15 and ChimeraX v.1.3 visualization packages. All data and python scripts used to generate the plots included in the manuscript are available in the following Github repository: https://github.com/Bharat-github6/Mechanical-activation-of-AT1-receptor. This Github repository also includes examples for how to estimate the free energy surfaces using the LDT bias with well-tempered metadynamics.

For manuscripts utilizing custom algorithms or software that are central to the research but not yet described in published literature, software must be made available to editors and reviewers. We strongly encourage code deposition in a community repository (e.g. GitHub). See the Nature Portfolio guidelines for submitting code & software for further information.

Data

Policy information about availability of data

All manuscripts must include a data availability statement. This statement should provide the following information, where applicable:

- Accession codes, unique identifiers, or web links for publicly available datasets
- A description of any restrictions on data availability
- For clinical datasets or third party data, please ensure that the statement adheres to our policy

All data and python scripts used to generate the plots included in the manuscript are available in the following Github repository: https://github.com/Bharat-github6/Mechanical-activation-of-AT1-receptor. This Github repository also includes examples for how to estimate the free energy surfaces using the LDT bias with well-tempered metadynamics. Any other data or simulation trajectories are available from the corresponding author upon reasonable request.

Human research participants

Policy information about studies involving human research participants and Sex and Gender in Research.

Reporting on sex and gender	N/A
Population characteristics	N/A
Recruitment	N/A
Ethics oversight	N/A

Note that full information on the approval of the study protocol must also be provided in the manuscript.

Field-specific reporting

Please select the one below that is the best fit for your research. If you are not sure, read the appropriate sections before making your selection.

☒ Life sciences ☐ Behavioural & social sciences ☐ Ecological, evolutionary & environmental sciences

For a reference copy of the document with all sections, see nature.com/documents/nr-reporting-summary-flat.pdf

Life sciences study design

All studies must disclose on these points even when the disclosure is negative.

Sample size	The length of the simulations were chosen in the 2 to 20 microsecond range to observe the molecular level fluctuations that occur at long timescales. These were determined based on the observed behavior as well as other published data.
Data exclusions	No data was excluded.
Replication	All simulations were run in duplicate. Additional enhanced-sampling simulations and Anton 2 simulations serve as further replicas.
Randomization	The behavior of the replicas was differentiated by generating random initial velocities of all particles.
Blinding	N/A

Reporting for specific materials, systems and methods

We require information from authors about some types of materials, experimental systems and methods used in many studies. Here, indicate whether each material, system or method listed is relevant to your study. If you are not sure if a list item applies to your research, read the appropriate section before selecting a response.

Materials & experimental systems

n/a	Involved in the study
☒	☐ Antibodies
☒	☐ Eukaryotic cell lines
☒	☐ Palaeontology and archaeology
☒	☐ Animals and other organisms
☒	☐ Clinical data
☒	☐ Dual use research of concern

Methods

n/a	Involved in the study
☒	☐ ChIP-seq
☒	☐ Flow cytometry
☒	☐ MRI-based neuroimaging
